# The Third Annual Announcement of the College of Medicine and Surgery of the University of Michigan

**Published:** 1852-08

**Authors:** 


*The Third Annual Announcement of the College of Medicine and Surgery of the University of Michigan.*

HAVING felt a deep interest in the success of the principle on which the Regents of Michigan have founded their school of medicine, we have never omitted to improve a fitting opportunity to speak a word in its favor, and we are sincerely glad to see by the present catalogue, unmistakeable evidences of its prosperity. Having uniformly felt and spoken thus, we were not a little surprised, on reading the present announcement, to find one or two paragraphs which certainly justify, if they do not demand, some comments.

The paragraphs that first arrested our attention, and caused a much more careful examination of the whole circular, were as follows, viz.:—"Others have reduced their fees, but have *not* enhanced their requisitions—an innovation, certainly, but one with which *those connected with this institution do not wish to be identified or confounded.*

"Fortunately, Michigan has the desire and ability to possess herself of the honor of being the pioneer in this important movement. The experiment has been tried, and the result has been unexpectedly satisfactory. The peculiar position of the College of Medicine and Surgery of the University of Michigan, *is such that it has been enabled fully to comply with the demands of the profession*, at the same time that it has sustained itself."

The first sentence here quoted is so evidently designed as a contemptuous fling at the Rush Medical College, and embodies so palpable an untruth, that the authors of it need blame no one but themselves if it provokes a full inquiry into the truth of the pretension so arrogantly set forth in the last part of the quotation, viz.:—that the position "of the College of Medicine and Surgery of the University of Michigan is such *that it has been enabled fully to comply with the demands of the profession.*"

Two enquiries will very naturally arise in the minds of the reader, viz.: First, what *are* "the demands of the profession" on our medical colleges; and second, in what manner has "the College of Medicine and Surgery of the University of Michigan" *fully complied* with these demands?

A full answer to the first of these enquiries can be found on page 74 of volume 1st of the Transactions of the American Medical Association; and is as follows, viz.:—

"Resolved, 1st. That it be recommended to all the colleges to extend the period employed in lecturing, from four to six months.

"2d. That no student shall become a candidate for the degree of M. D., unless he shall have devoted *three entire years to the study of medicine*, including the time allotted to attendance on lectures.

"3d. That the candidate shall have attended two full courses of lectures, that he shall be twenty-one years of age, and in all cases shall produce the certificate of his preceptor, to prove when he commenced his studies.

"4th. That the certificate of no preceptor shall be received who is avowedly and notoriously an irregular practitioner, whether he shall possess the degree of M. D. or not.

"5th. That the several branches of medical education already named in the body of this report, be taught in all the colleges and that the *number of professors be increased to seven.*

"6th. That it be required of candidates that they *shall have steadily devoted three months to dissections.*

"7th. That it is incumbent upon preceptors to avail themselves of every opportunity to impart clinical instruction to their pupils; and upon *medical colleges to require candidates for graduation to show that they have attended on hospital practice, for one session*, whenever it can be accomplished, for the advancement of the same end."

Such are the formal *demands* of the profession on the medical colleges, as deliberately adopted by the National Medical Convention in 1847; and which have been re-affirmed by almost every annual meeting of the American Medical Association since that time. Indeed, so much importance was attached to the 7th *demand* enumerated, that at the largest meeting of the Association ever convened, (the meeting in Boston, May 1849,) it was almost unanimously re-affirmed in the following explicit language, viz.:—

"Resolved, That the Association *does not sanction or recognize 'college clinics'* as *substitutes* for *hospital clinical instruction;* and *that the medical colleges be again advised to insist,* in all instances where it is practicable, on the regular attendance of their pupils, *during a period of six months, upon the treatment of patients in a properly conducted hospital,* or other suitable institution devoted to the reception and cure of the sick.'

Now, the following quotation from the Annual Announcement before us, will enable every reader to see how far, and in what manner, our friends in Michigan have *complied with the demands* of the profession.

"To be admitted to the degree of Doctor of Medicine, the student must exhibit evidence of having pursued the study of medicine and surgery for the term of *three years,* with some respectable practitioner of medicine (including the lecture terms); must have attended two full courses of lectures, the last of which must have been in the College of Medicine and Surgery of the University of Michigan, and the previous one in this or some other respectable medical institution; must have been engaged in the study of practical anatomy; must be twenty-one years of age; must have submitted to the faculty a thesis, composed and written by himself, on some medical topic, and have passed an examination at the close of the term satisfactory to the faculty. To encourage a higher grade of preliminary acquirement, *an allowance of one year from the term of study* is made in favor of graduates of the College of Science and Arts, and of other respectable literary colleges."

Can anything be found here complying *fully* with the 2d, 5th, 6th, and 7th "*demands*" of the profession as already quoted? Does the phrase, "must have been engaged in the study of practical anatomy," ensure the devotion of "*three months to dissections?*" Does the admission of students to the honor of the Doctorate, after only *two years' study,* ensure the devotion "*of three entire years to the study of medicine?*" Does the entire silence in regard to *clinical instruction,* "require candidates for graduation to show *that they have attended on hospital practice for one session?*" These questions are neither impertinent nor nappropriate at the present time. For several years the subject of medical education has engaged the earnest attention of the profession. The medical schools have been severely censured for not adopting and exacting a higher standard of requirements before admitting students to an examination for the degree of M. D. Some have proposed one remedy and some another. For ourselves, we fully adopted the sentiments of the venerable Alexander H. Stevens, as expressed in his address before the New York State Medical Society, and which have been nominally acted on by the Regents of the University of Michigan. These sentiments were briefly and substantially; first, to remove as far as possible the *pecuniary expense* incurred by the student, for the purpose of enabling him to devote a *longer time* to study; second, the rendering the professors or teachers as *little dependent as possible* on the mere *number* of students under their instruction, by paying them fixed salaries, that they might *feel able and free to rigidly require a high standard* of medical attainment before granting the honors of the schools.

When the Regents of the State of Michigan organized the medical department of their university, removed all *pecuniary* burden from the student by making the *lectures free*, and paid the professors fixed salaries from their ample university fund, we naturally looked to them with much confidence for a practical illustration of the correctness of the sentiments we had imbibed.

Our first disappointment, was in their accepting a location where the all-important subject of clinical or hospital instruction must be entirely excluded.

Our second, and greater disappointment, was founded in the present "*Announcement*," in which, after claiming a class of 157 matriculants, and a success "unexpectedly satisfactory," we not only find all *clinical* instruction omitted, but we find no positive requirement of even "*three months' dissections*," and a miserable *discount* of *one-third* of the whole period usually required for medical studies, in favor of those who could manage to get the title of A. B., from some one of the thousand literary institutions of our country.

Where is the necessity of thus narrowing down the period of medical study? We are told, rather pompously, that "the *peculiar position* of the College of Medicine and Surgery of the University of Michigan, *is such*, that it has been *enabled fully to comply with the demands* of the profession." Then why *hire* the student to get certain preliminary attainments, at the enormous *price* of *one-third* of the short period of three years' medical pupilage? What part of the profession have *demanded* any such sacrifice? When the profession demanded of the colleges a longer lecture term and a less number of lectures per day, it was not for the purpose of merely giving the student more time to read books, which he could as well do in his closet at home; but it was for the express purpose of enabling him to devote a part of every day to the important work of *dissections* and *clinical observations* by the bed-side of the sick, where the *principles taught*, should be tested practically by the *teacher*. When endowments were asked sufficient to render the professors in the colleges pecuniarily independent of the income derived from students, it was for the double purpose of taking away from the student the oft-repeated plea that he was pecuniarily unable to study long enough or attend courses of lectures enough, and of placing the professors in a position that would enable them uniformly to demand from the student such attainments, both preliminary and medical, as would make him useful to the community and an honor to the profession. In the correctness of these sentiments we still have the most implicit confidence. But until the medical department of the University of Michigan carries them more fully into practice, than she promises to in the present "*Announcement*," we shall be constrained to join heartily in their wish not to have their faculty, in any wise, "*identified* or *confounded*," with us. N. S. D.

